# Effect of Sodium Azide on Quantitative and Qualitative Stem Traits in the M2 Generation of Ethiopian Sesame (*Sesamum indicum* L.) Genotypes

**DOI:** 10.1155/2021/6660711

**Published:** 2021-04-30

**Authors:** Micheale Yifter Weldemichael, Yemane Tsehaye Baryatsion, Desta Berhe Sbhatu, Girmay Gebresamuel Abraha, Hagos Mohammedseid Juhar, Abraha Birhan Kassa, Fiseha Baraki Sibhatu, Hailay Mehari Gebremedhn, Tesfakiros Semere Gebrelibanos, Mohammed Mebrahtu Mossa, Mullubrhan Mekonen Gebru, Birhanu Kahsay Meresa, Medhin Teklay, Birhanu Debesay Berhe, Haftay Abadi Gebru

**Affiliations:** ^1^Mekelle University, P.O. Box 231, Mekelle, Tigrai, Ethiopia; ^2^Humera Agricultural Research Center, P.O. Box 523, Humera, Tigrai, Ethiopia; ^3^Tigrai Biotechnology Center Pvt. Ltd. Co., P.O. Box 223, Mekelle, Tigrai, Ethiopia

## Abstract

The emerging oilseed crop *Sesamum indicum,* also known as the queen of oilseeds, is being grown globally for its oil content for medicinal and nutritional values. One of the key challenges of sesame cultivation is its low productivity. In the present study, sodium azide (NaN_3_) was used as a chemical mutagen. The aim of this study was to examine the effect of NaN_3_ on quantitative and qualitative stem traits in the M2 generation of Ethiopian sesame (*Sesamum indicum* L.) genotypes. Seeds of fourteen sesame genotypes were used in this study and germinated and grown under greenhouse conditions. Different qualitative and quantitative data were collected and analyzed. Traits such as plant height, ground distance to first distance, and internode length were significantly affected by NaN_3_ treatment. The highest plant height was recorded in the control on Humera 1 and Baha Necho genotypes, while the lowest was observed on Setit 2 and Hirhir treated with the chemical. The highest ground distance to the first branch was observed in Gumero, while the least ground distance was recorded in Setit 1 in the treated and control genotypes, respectively. The best internode length was recorded on Setit 2 and ADI in the control, while the lowest internode length was observed in Setit 1 genotype treated with sodium azide. Genotypes such as ACC44, ADI, Baha Necho, Borkena, Gonder 1, and Setit 1 treated with NaN_3_ have showed glabrous type of stem hairiness. All the fourteen genotypes (both treated and control) were clustered into four groups. In conclusion, we observed a highly significant variation among the genotypes due the effect of the chemical and genotypes themselves. Hence, this report would create more genetic diversity for further sesame genetic research improvements.

## 1. Introduction

Sesame (*Sesamum indicum* L., 2*n* = 26) is one of the ancient oilseed crops, cultivated in the arid and semiarid regions of Africa, Asia, and South America [1, 2]. Sesame seed is an important oilseed crop which has high nutrition and oil quality and pharmaceutical and cosmetic uses [3]. Sesame seeds are rich in oil (50–55%), proteins (18–20%), carbohydrate (13.4–25.0%), and digestible fiber (9.8%) [4]. Sesame seed has numerous applications for nutritional, pharmacological, and industrial uses due to its high-quality oil and referred as the “queen of oilseeds” [2, 5, 6]. Sesame seed is also rich in vitamins such as vitamin E, vitamin C, vitamin A, thiamine (B1), riboflavin (B2), niacin (B3), pyridoxin (B6), and folate (B9) and minerals including calcium (1.35%), potassium (0.67%), pantothenic acid (0.60%), phosphorous, iron, magnesium, zinc, and vitamin B1 [7]. Along with enhancing the nutritional importance of the crop, enhancing the yield is one of the key objectives of sesame breeding and production [8, 9].

The yield of sesame is affected by several factors such as seed number and plant height [10], capsule length [11], seed number per capsule and capsule length [12], capsule number [13], determinate growth [14], internode length and dwarfing [15], and controlling the branching habit [16]. Besides, yield is also determined by plant height and other several traits [17]. Plant height and internode length are among the most important agronomic traits for sesame production [18]. Semidwarfing and dwarfing mutations can affect the plant height and contribute to high lodging resistance and yield as the maturity and harvest index are always stimulated in mutants of dwarf and semidwarf genotypes [19]. A dwarf mutant with a short internode length, named Dw607, was induced from variety Yuzhi 11 by EMS mutagenesis [10]. The semidwarf and dwarf phenotypes have been cloned from wheat [11, 12], maize [13], rice [14], soybean [15], *Brachypodium distachyon* [16], and *Gossypium arboretum* [20]. Moreover, breeding dwarf or semidwarf rice and wheat varieties is carried out to accomplish an enhanced harvest index and upgraded adaptation to the irrigated and convenient environments generally inhabited by taller plants [21–23]. Due to its high plant height, sesame has low harvest index, low yielding capacity, and lodging and is susceptible to abiotic and biotic stresses [24]. Accordingly, it is very important to reduce plant height significantly and improve lodging resistance so as to increase its production [24].

Determinate growth habit through mutation induction is one important prerequisite for developing sesame cultivars suited to modern planting systems combined with mechanized harvesting. Nowadays, many mutagen-induced high-yielding sesame genotypes are available [25]. Chemical mutagenesis is considered as an effective means in improving the quality and yield traits of crop plants [26]. Among many alkylating chemical mutagens, ethyl methane sulphonate (EMS), hydrazine hydrate, and sodium azide (SA) are commonly used in many crop species [27]. These chemicals improve sesame yield through the development of cultivars with determinate growth habits and modified plant morphology, improved seed retention, synchronous maturity and earliness, resistance to diseases, male sterility, larger seed size, desired seed coat color, higher oil content, and modified fatty acid composition [28–31]. Indeterminate growth habit, characterized by continuous flowering and nonuniform capsule ripening is, however, the most critical challenge for sesame breeders [29].

In a variety of crop improvement programs, sodium azide had been and is still being used as potent mutagens. It is a well-known inhibitor of cellular respiratory processes in living cells [32] and has proven to be an effective mutagen in many crop species such as barley [33–35], tomato [36], oat species *Avena longiglumis* [37], and rice [38]. Up to now, mutation breeding of Ethiopian sesame genotypes targeted to stem-related traits has not yet been reported. In this regard, it is vital to grow mutants with stem traits that have a potential for production of new mutants with better desirable traits to accelerate the breeding program and improve sesame genotypes. Therefore, the present study was investigated to examine the effect of sodium azide on quantitative and qualitative stem traits in the M2 generation of fourteen Ethiopian sesame (*Sesamum indicum* L.) genotypes.

## 2. Materials and Methods

### 2.1. Plant Materials

Seeds of fourteen sesame genotypes, comprising five local collections collected from the Ethiopian Biodiversity Institute (EBI) (namely, Aberghele, Bounji, Gumero, Hirhir, and Zeri Tesfay), eight released varieties from different research centers in Ethiopia (namely, ACC44, Baha Necho, Baha Zeyit, Borkena, Gondar 1, Humera 1, Setit 1, and Setit 2), and an introduced variety (ADI) from Israel, were used (Table 1). All these genotypes were available at and collected from the Humera Agricultural Research Center (HuARC), Western Tigrai, Ethiopia. The seeds were normally shaped, disease-free, and dry.

### 2.2. Methods

#### 2.2.1. Sterilization

The seeds were initially washed with running tap water for 20 min, soaked in 3% of teepol detergent solution for 5 min, and rinsed with distilled water. They were then disinfected with 70% ethanol for 45 sec at room temperature and rinsed with sterile distilled water three times. Then, the seeds were treated with concentrated H_2_SO_4_ for 15–20 min and spread onto Petri-dishes. The seeds were sterilized in a closed desiccator for 60 min with 20 ml commercial bleach mixed with 10 ml glacial acetic acid [39]. Following sterilization, the seeds were soaked in sterile water for 16 hrs.

#### 2.2.2. Treatment with Sodium Azide

The seeds were presoaked in cold tap water (4°C) for 24 hrs and then soaked in 0.75% sodium azide (NaN_3_) solution (Kiran Light Laboratories, Mumbai-400002, India) with Sörenson phosphate (Sigma-Aldrich, Munich, Germany) as a buffer adjusted to pH = 3 and H_3_PO_4_ (Sigma-Aldrich, Munich, Germany) for 4 hrs with constant shaking at 20 rpm at 18–24°C. The seeds were then rinsed seven times with sterile water. After the final wash, 40 ml of sterile water was added for each genotype and left overnight at the magenta culture bottles (cap material: pp H200MK Reliance food-grade transparent autoclavable, type: glass jars, color: transparent, and brand name: HnG, India). The next day, the seeds were drained and air-dried. Excess chemical residue was removed by washing the seeds in running tap water for at least 4 hrs. Untreated seeds were used as control. After washing, both the control and treated seeds were planted in well-prepared beds in greenhouse to get M1 seeds. Finally, healthy, clean, and good-looking seeds were collected from the M1 plants and planted to generate M2 plants.

#### 2.2.3. Greenhouse Evaluation of M2 Lines

Ninety seeds of each of the M2 lines were planted under greenhouse conditions on 2 m × 2 m plots (beds) with spacing of 40 cm between rows and 10 cm between plants using a completely randomized design (CRD) with three replications. All the necessary production practices such as irrigation, fertilization, and weed management were carried out as required during the growth period of the plants. All the required quantitative and qualitative data were collected from five plants of each of the 14 sesame M2 lines at maturity. Recorded quantitative data include (a) ground distance to the first branch (GDFB), (b) internodes length (IL), and (c) plant height (PH). Likewise, recorded qualitative data were (a) main stem color (MSC), (b) stem hairiness (SH), and (c) stem branch (SB) (Table 2). Plant height was measured from the base of the shoot to the tip the upper leaf in centimeters using a ruler.

#### 2.2.4. Data Analyses

All the data collected were subjected to analysis of variance (ANOVA) using GenStat 18 software [40]. The comparisons of means were carried out using Duncan's Multiple Range Test (DMRT) at a significance level of *p* ≤ 0.01 [41].

## 3. Results and Discussion

The results presented that all the traits were significantly affected by sodium azide treatment. This study was conducted for the production of mutants having unique characters related to enhanced yield and quality of grains from the Ethiopian genotypes in relation to plant height and related traits as a main trait for yield improvement.

### 3.1. Effects of NaN_3_ on Quantitative Traits of M2 Plants

The ANOVA result showed that the NaH_3_-treated M1 seeds resulted in varied effects on the various traits of the M2 plants of the 14 genotypes. The interaction effects (NaH_3_ on the genotypes) on quantitative traits were significantly different from each other for different characters. Thus, the concentration of NaN_3_ and the genotypes (interaction effect) showed highly significant effect on the various quantitative data including ground distance to the first branch (GDFB), internode length (IL), and plant height at *p* ≤ 0.01 (Table 3).

#### 3.1.1. Plant Height

**Figure 1:**
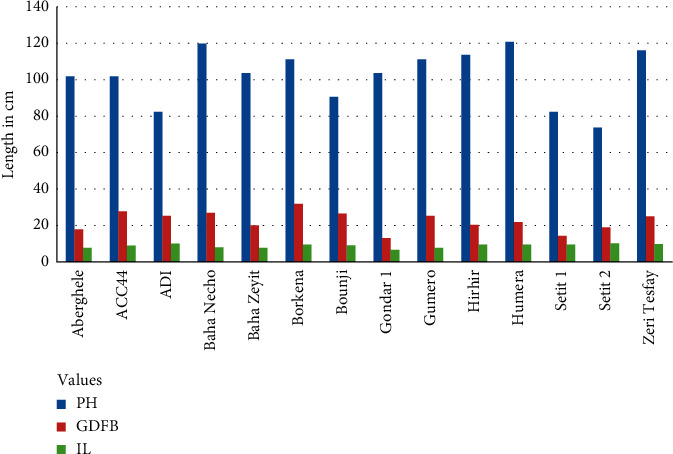
The responses of different genotypes on plant height (PH), ground distance to the first branch (GDFB), and internode length (IL) at the control (without sodium azide).

**Figure 2:**
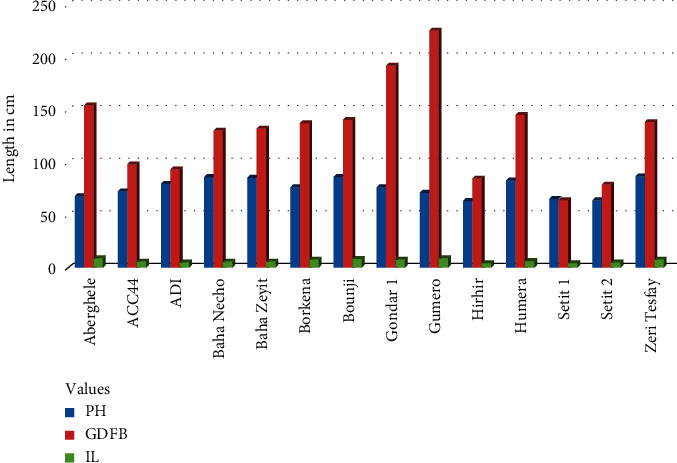
Effects of sodium azide supplements and genotypes on plant height (PH), ground distance to the first branch (GDFB), and internode length (IL).

The highest plant height was recorded in the control on Humera 1 (120.68 cm) and Baha Necho (119.35 cm) genotypes (Figure 1), while the lowest was observed on Setit 2 (64.59 cm) treated with the chemical (Figure 2) (Table 4). Similar results have reported that the plant height of the mutant plants was reduced from 170 to 110 cm [10]. Besides, the plant height of *dw607* (*dwf1*) significantly declined from 176.00 to 118.25 cm [42]. A similar study was conducted using sodium azide on quantitative traits of sesame [43]. Their findings reported that the maximum plant height at maturity was 66 cm while the minimum plant height was 55 cm at 0.02% and 0.05% sodium azide concentration, respectively. In addition, sesame plant height reduction by mutation via sodium azide was also reported [44, 45]. In this experiment, genotypes treated with chemicals have reduced their plant height by almost half, i.e., from 120.68 cm to 64.59 cm. To the best of our knowledge, the sesame cultivars cultivated formerly have had indeterminate growth habits, thus causing the nonuniform ripening of the capsules and resulting in seed loss at harvest. Mutation breeding is very crucial to produce genotypes with important traits such as production of determinate growth habit, synchronous uniform flowering, and homogeneous maturity in a short period of time, and this has been practiced for the development and domestication of many crop plants [29]. Accordingly, the maximum plant height acceptable for all harvest implements was 150 cm and lower plants were more preferable [8]. In this regard, higher plant height is often susceptible to lodging due to a strong wind or Hurricane. Similar results have been reported on reduction of plant height after treatment of seed with sodium azide at low pH value [46–48]. Similarly, plant height decides the plant architecture and contributes to the yield of sesame [49]. Hence, tall plant types are usually sensitive to lodging, associated with shy branching and show less fruiting density which is unfavorable for mechanized harvesting and undesirable for high yield. Dropping plant height, as a means of enlightening lodging resistance is, therefore, very significant for sesame breeders.

The plant height at maturity was reduced when treated with concentration of sodium azide as reported in black gram [50] and in cowpea [51]. In addition, the work in [52] reported a decrease in seedling plant heights and root length of popcorn (*Zea mays* var. Praecox Sturt.) with increased gamma rays and thermal neutrons. Similar results had been reported by Singh et al. [53] who stated that seedling survival was dependent on dissimilar concentrations of sodium azide. Thus, reduced plant height, increased yield, and early flowering are important targets for sesame genetic improvement. In parallel, a dwarf mutant *dw607* (*dwf1* type) has been created using ethyl methanesulfonate (EMS) mutagenesis in [54]. Compared with the wild type (Yuzhi 11), the plant height of *dw607* declined by more than 40%, and the internode length reduced about 50%. More importantly, yield of dwarf varieties derived from *dw607* significantly increased under rich fertilizers and water culture conditions [42]. Thus, creation of a dwarf mutant supplies a valuable material for exploring the genetic mechanism of plant height trait and dwarf variety breeding in sesame. Hence, the result of this study often set up reducing plant height which is important for sesame breeding.

#### 3.1.2. Ground Distance to the First Branch

The highest ground distance to the first branch was observed in the treated Gumero (44.76 cm) and Gondar 1 (38.16 cm) genotypes (Figure 2). On the other hand, the least ground distance was recorded in the treated genotypes including Setit 1, Hirhir, ADI, ACC44, Setit 2 and Setit 1 (Figure 2), and Gondar 1 in the control (Figure 1) (Table 4). Ground distance to the first branch is affected by genotypes (treated and control) and its environment such as moisture, time of planting, light quality, and population besides genotypes. Slow developmental rhythm in the crop is always accompanied with late flowering as it is not adapted to relatively long days. Mutants such as thick leaf lines are most preferred as they possess superior agronomic traits such as more branches per plant, more capsules on the main axis, longer distance from base to first branching, more capsules per plant, higher seed yield and higher seed protein content as compared to the parent variety [55]. Mutations that changed M2 sesame leaf morphology to desirable agronomic traits were induced by ethyl methane sulphonate (EMS) [56].

#### 3.1.3. Internode Length

The lengths between two successive leaves per plant were measured from five plants per each genotype using a meter rule. Internode length (IL) was measured as an average of internode distances on the same stalk from five plants of the same genotype. The best IL was recorded from Setit 2 (11.47 cm) and ADI (11.13 cm) in the control (Figure 1), while the lowest IL was observed in Setit 1 genotype treated with sodium azide (Figure 2) (Table 4). Similarly, a dwarf mutant with a short internode length, named Dw607, was induced from variety Yuzhi 11 by EMS mutagenesis, and the internode lengths of the mutant plants decreased from 6.0 to 8.4 to 3.5–4.0 cm [10]. This result is in contradiction to the work of Birara et al. [43] who studied mutation breeding using NaN_3_ on quantitative traits of sesame, and their findings reported that there was no significant difference observed in internode length of various concentrations of mutagen treatments including the control.

Several gamma-irradiated mutant lines with important traits such as fine reduced internode length, reduced stolon length, and leaf texture were described in St. Augustine grass and in bermudagrass [57–59]. The short internode length and dwarfing trait in mutant dw607 is reported to be controlled by a recessive gene allele (Sidwf1) [42]. Previous studies [60, 61] have reported the role of chemical mutagens in improving genetic diversity in higher plants. Mutants such as barley, cotton, rice, peanuts, wheat, and beans produced facilitate the identification, isolation, and cloning of genes used in designing crops with improved quality and yield traits [62]. Therefore, the artificial mutation is a practical means to achieve genetic improvement in crop species and is performed with physical and/or chemical mutagens that enlarge the mutation frequency, when compared to the spontaneous occurrence.

### 3.2. Effects of NaN_3_ on Qualitative Traits of M2 Plants

This study also investigated the effects of 0.75% NaN_3_ on stem hairiness (Table 5). The responses of the traits to the 0.75% NaN_3_ treatment are described below.

#### 3.2.1. Steam Hairiness

Qualitative analysis for stem hairiness indicated a glabrous type for 100% of the treated genotypes of ACC44, ADI, Baha Necho, Borkena, Gonder 1, and Setit 1, whereas those genotypes used as control had weak/sparse stem hairiness. On the other hand, application of the treatment has changed the steam hairiness of Hirhir and Humera 1 genotypes from glabrous stem hairiness to weak/sparse.

## 4. Grouping and Calculating Mahalanobis Distance

### 4.1. Grouping of Fourteen Sesame Genotypes

**Figure 3:**
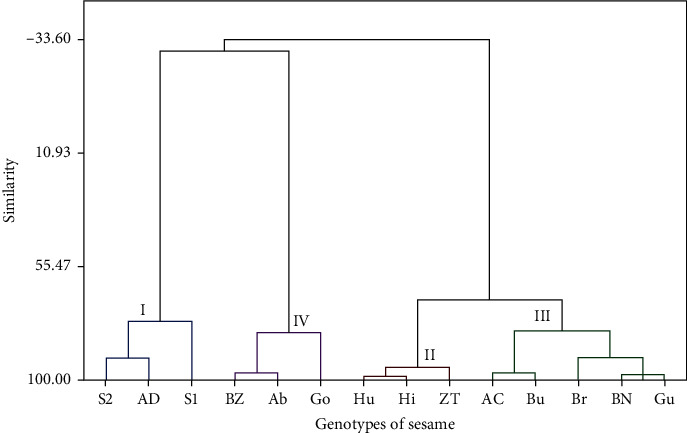
Dendrogram using Ward's method based on the dissimilarity matrix of 14 sesame genotypes in the control. Ab = Aberghele, Ac = ACC44, AD = ADI, BN = Baha Necho, BZ = Baha Zeyit, Br = Borkena, Bu = Bounji, Go = Gondar 1, Gu = Gumero, Hi = Hirhir, Hu = Humera 1, S1 = Setit 1, S2 = Setit 2, and ZT = Zeri Tesfay.

**Figure 4:**
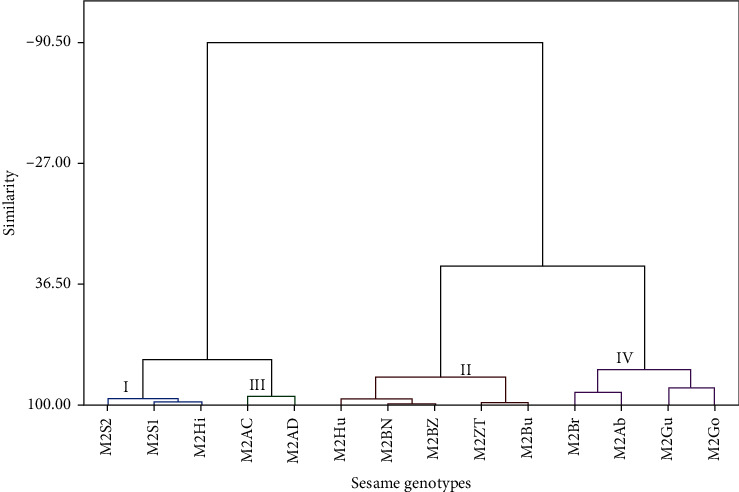
Dendrogram using Ward's method based on the dissimilarity matrix of 14 sesame genotypes treated with NaN_3_. M2Ab = mutant Aberghele, M2Ac = mutant ACC44, M2AD = mutant ADI, M2BN = mutant Baha Necho, M2BZ = mutant Baha Zeyit, M2Br = mutant Borkena, M2Bu = mutant Bounji, M2Go = mutant Gondar 1, M2Gu = mutant Gumero, M2Hi = mutant Hirhir, M2Hu = mutant Humera 1, M2S1 = mutant Setit 1, M2S2 = mutant Setit 2, and M2ZT = mutant Zeri Tesfay.

The 14 genotypes were clustered into four distinct groups both in the control and treated genotypes (Figures 3 and 4). Cluster mean values for each trait in the control are given in Table 6. Fourteen genotypes were clustered into four distinct groups (Figure 3). Cluster I contains three genotypes including S1 and S2 which are research-improved genotypes released from the Humera Agricultural Research Center and AD which is imported from Israel. They are characterized by a relatively moderate mean of ground distance to the first branch (20.44), lower mean of plant height (80.33), and higher mean of internode length (10.78). Cluster II, on the other hand, includes three genotypes (i.e., Hu, Hi, and ZT). These genotypes had the highest mean number of plant height (117.33), relatively higher mean ground distance to the first branch, and internode length 23.11 and 10.33, respectively. Cluster III has five genotypes, namely, Ac, Bu, Br, BN, and Gu. Three genotypes, namely, Ac, Br, and BN, are research-improved genotypes with the exception of Bu and Gu, which are locally adapted genotypes. The genotypes in this cluster are characterized by the highest ground distance to the first branch (28.40) and relatively higher plant height (107.07), as well as internode length (9.87). Finally, cluster IV includes three genotypes, namely, BZ, Ab, and Go. These genotypes had lower mean number of ground distance to the first branch and internode length (17.89 and 8.22, respectively) and relatively moderate mean plant height (103.22) (Table 6). The genotype grouping observed from the cluster analysis was further confirmed by the Mahalanobis distance analysis among clusters (Table 7). The distance values range from 27.47 (between clusters I and II) to 26.53 (between clusters II and III), and all the distance values were significantly different from each other (*p* ≤ 0.01). The significant difference among clusters as depicted by the Mahalanobis distance would have a breeding implication in sesame improvement programs in Tigrai.

### 4.2. Grouping of Fourteen Sesame Genotypes Treated with NaN_3_

The cluster mean values for each trait in the treated genotypes are given in Table 8. The 14 genotypes were grouped into four distinct clusters (Figure 4). Cluster I contains three genotypes, namely, M2S1, M2S2, and M2Hi. These are characterized by the lowest mean number of plant height (64.67), lower mean ground distance to the first branch (15.27), and lower mean number of internode length (4.87). Cluster II includes five genotypes, namely, M2Hu, M2BN, M2BZ, M2ZT, and M2Bu. These genotypes had the highest mean number of plant height (86.16) and relatively higher ground distance to the first branch (27.58) and internode length (7.24). Cluster III has two genotypes, namely, M2Ac and M2AD. The genotypes in this cluster are characterized by the moderate mean number of plant height (76.8), mean ground distance to the first branch (19.30), and internode length (5.40). Finally, cluster IV includes four genotypes, namely, M2Br, M2Ab, M2Gu, and M2Go. These genotypes had higher mean number of internode node length (8.7), higher mean number of ground distance to the first branch (35.60), and moderate mean number of plant height (73.10) (Table 8). The genotype grouping observed from the cluster analysis was further confirmed by the Mahalanobis distance analysis among clusters (Table 9). The distance values range from 76.91 (between clusters I and II) to 20.58 (between clusters II and III).

To sum up, the distance values were tested for their significance both at 1% and 5% probability for all the control and treated plants. The distances between groups indicate that some are distinct from others. The minimum distance was found between cluster III and IV (*D*^2^ = 12.03) and between cluster II and IV (*D*^2^ = 18.09) in the control and treated plants, respectively. The highest distance was detected between cluster II and IV in the control plants and between cluster I and II in the treated plants. This suggests that cluster II and IV (in control plants) and cluster I and II (in treated plants) are the most distinct from others. In other words, there is wide variability among the genotypes that are under these clusters. These distinct clusters have breeding implications in that crossing by choosing parents from these clusters may bring variability in the progeny. On the other hand, crossing between clusters that have lower distance, in the case of clusters III and IV in control plants, may not produce desirable recombinants as they are similar genotypes. The genotypes in the same cluster can be mixed altogether and can be treated as one genotype as they are assumed to have similar characteristics. These could be sown together in an approach called evolutionary breeding so that we can get potential genotypes that are adaptable to a wide range of biotic and abiotic stress tolerant and adaptable to the ever-increasing climate change [63, 64].

### 4.3. Grouping of Sesame Genotypes in Combination with Mutants and Their Control Lines

**Figure 5:**
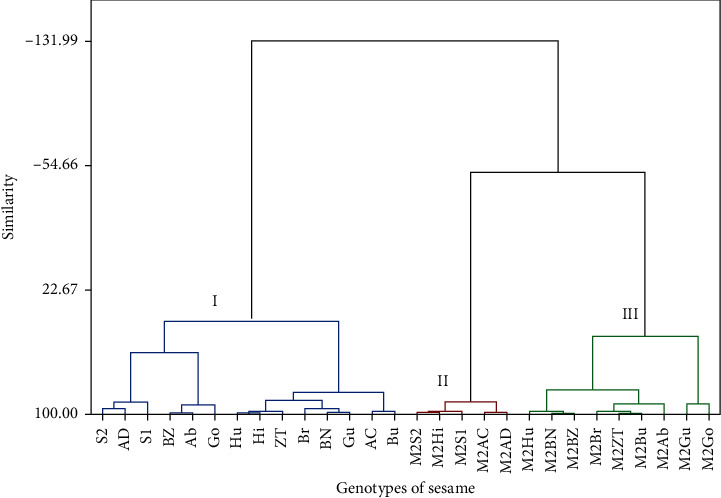
Dendrogram using Ward's method based on the dissimilarity matrix of 14 sesame genotypes treated with NaN_3_ and their control parents. Ab = Aberghele, Ac = ACC44, AD = ADI, BN = Baha Necho, BZ = Baha Zeyit, Br = Borkena, Bu = Bounji, Go = Gondar 1, Gu = Gumero, Hi = Hirhir, Hu = Humera 1, S1 = Setit 1, S2 = Setit 2, ZT = Zeri Tesfay. M2Ab = Mutant Aberghele, M2AC = Mutan ACC44, M2AD = Mutan ADI, M2BN = Mutan Baha Necho, M2BZ = Mutan Baha Zeyit, M2Br = Mutan Borkena, M2Bu = Mutan Bounji, M2Go = Mutan Gondar 1, M2Gu = Mutan Gumero, M2Hi = Mutan Hirhir, M2Hu = Mutan Humera 1, M2S1 = Mutan Setit 1, M2S2 = Mutan Setit 2, and M2ZT = Mutan Zeri Tesfay.

The combination of mutants with their control genotypes were clustered into three distinct groups (cluster I contains control genotypes and cluster II and III contain mutated genotypes) (Figure 5). Cluster I contains all control genotypes, namely, Ab, Ac, AD, BN, BZ, Br, Bu, Go, Gu, Hi, Hu, S1, S2, and ZT. They are characterized by the highest mean number of plant height and internode length (102.71 and 9.810, respectively) and moderate ground distance to the first branch (23.31). Cluster II, on the other hand, includes five genotypes (i.e., M2S2, M2Hi, M2S1, M2Ac, and M2AD). These genotypes had the lowest mean number of plant height, ground distance to the first branch, and internode length (69.52, 16.88, and 5.080, respectively). Finally, cluster III includes nine genotypes, namely, M2Hu, M2BN, M2BZ, M2Br, M2ZT, M2Bu, M2Ab, M2Gu, and M2Go. These genotypes had the highest mean number of ground distance to the first branch (31.14) and relatively moderate mean number of plant height and internode length (80.36 and 7.889, respectively) (Table 10). This finding which is associated with reducing plant height would play a vital role in facilitating mechanized harvesting and thereby increasing yield of sesame. The genotype grouping observed from the cluster analysis was further confirmed by the Mahalanobis distance analysis among clusters (Table 11). The distance values range from 48.58 (between clusters I and II) to 11.71 (between clusters II and III), and all the distance values were significantly different from each other (*p* ≤ 0.01).

## 5. Conclusions

Mutation breeding is one of the popular breeding methodologies to create nonexisting variations. Chemical mutagenesis has proven to be extremely useful to create new allelic variants that can be used in functional genomic studies and/or plant breeding. Sodium azide is a potential chemical mutagen facilitating crop improvement. The results showed that all the traits were significantly affected by sodium azide treatment. Comparison of both qualitative and quantitative traits of the mutant seeds of the fourteen varieties with those of the controls showed a highly significant difference (*p* < 0.01). Significant differences were observed between the mutant and the control for almost all of the morphological features/traits. Sodium azide at the concentration of 0.75% played the most important role in improving the quantitative and qualitative traits of *S. indicum* L. The highest plant height was recorded in the control on Humera 1 and Baha Necho genotypes, while the lowest was observed on Hirhir and Setit 2. Therefore, NaN_3_ can produce a range of phenotypes and provide an in-depth characterization of genetic function. To improve the yield parameters of sesame, selection towards nonbranching and determinate growth will be beneficial, and sodium azide can be used as a potential mutagen at the specific concentrations for realizing earliness in sesame crop. More research should also be conducted concurrently with other traits such as disease and insect pest resistance, drought, salinity, and osmotic tolerance to improve sesame production. Furthermore, research should be conducted with molecular markers which can be used to determine detail genetic diversity.

## Figures and Tables

**Table 1:** List of 14 sesame genotypes with their genotype code, mutant code, pedigree, and other traits.

Genotype name	Genotype code	Mutants code^*∗*^	Local/ improved	Year of release	Pedigree name	Average grain yield (kg·Ha^−1^)	Growth habit	Oil content in %	Seed color	Released
Aberghele	Ab	M2Ab	Local	—	—	—	Indeterminate	—	Red	—
ACC44	AC	M2AC	Improved	2013	Acc0047	700–800	Indeterminate	50.4	White	SiARC
ADI	AD	M2AD	Imported	1993	Accadd	1700	Indeterminate	40–58	White	Israel
Baha Necho	BN	M2BN	Improved	2016	Acc-EW-012 (5)	1200	Indeterminate	52	White	HU
Baha Zeyit	BZ	M2BZ	Improved	2016	Acc-EW-012 (3)	1300	Indeterminate	56	Light gray	HU
Borkena	Br	M2Br	Improved	2007	Acc.003	600–800	Indeterminate	47–48	Brown	HuARC
Bounji	Bu	M2Bu	Local	—	—	—	Indeterminate	—	Light gray	—
Gondar 1	Go	M2Go	Improved	2016	Acc.ba002	500–900	Indeterminate	50	White	GoARC
Gumero	Gu	M2Gu	Local	—	—	—	Indeterminate	—	White	—
Hirhir	Hi	M2Hi	Local	—	—	—	Indeterminate	—	Hirhir	—
Humera 1	Hu	M2Hu	Improved	2011	Acc038 sel 1	590–900	Indeterminate	54.56	White	HuARC
Setit 1	S1	M2S1	Improved	2011	Col sel p#1	620–1000	Indeterminate	52.54	White	HuARC
Setit 2	S2	M2S2	Improved	2016	J-03	913	Indeterminate	53.77	White	HuARC
Zeri Tesfay	ZT	M2ZT	Local	—	—	—	Indeterminate	—	White	—

Source: MoARD, crop variety register book 2010–2017. Ab2 = Aberghele, AC2 = ACC44, AD2 = ADI, BN2=Baha Necho, BZ2 = Baha Zeyit, Br2=Borkena, Bu2 = Bounji, Go2 = Gondar 1, Gu2 = Gumero, Hi2 = Hirhir, Hu2 = Humera 1, S1 = Setit 1, S2 = Setit 2, ZT = Zeri Tesfay. SiARC = Sirinka Agricultural Research Center, HU = Haramara University, GoARC = Gondar Agricultural Research Center, ^*∗*^M2 = mutant 2.

**Table 2:** List of studied qualitative traits of M2 lines.

(1) Main stem color (MSC): 1 = green; 2 = yellow; 3 = purplish green; 4 = purple; 5 = others
(2) Stem hairiness (SH): 1 = glabrous; 2 = weak or sparse; 3 = medium; 4 = strong of profuse
(3) Stem branch (SB): 1 = opposite; 2 = alternate; 3 = ternate; 4 = mixed

**Table 3:** Analysis of variance for various characters in the M2 generation of sesame.

Source of variation	d.f.	PH	GDFB	IL
Var	13	670.8^*∗∗*^	288.77^*∗∗*^	7.343^ns^
Treatment	1	13885.7^*∗∗*^	51.07^ns^	207.429v
Lin	1	13885.7^*∗∗*^	51.07^ns^	207.429^*∗∗*^
Var. treatment	13	368.4^*∗*^	184.85^*∗∗*^	10.890^*∗∗*^
Var. lin	13	368.4^*∗*^	184.85^*∗∗*^	10.890^*∗∗*^
Residual	84	174.6	42.30	4.012

PH = plant height, GDFB = ground distance to the first branch, IL=internode length. ^*∗∗*^*p* ≤ 0.01; ^*∗*^*p* ≤ 0.05; ns: nonsignificant.

**Table 4:** Interaction effects of sodium azide supplements and genotypes on different quantitative traits of plant height.

Genotypes	PH		GDFB		IL	
	Control	Treated	Control	Treated	Control	Treated
Aberghele	101.35^a–h^	68.19^fgh^	19.40^bcd^	30.56^abc^	9.118^a–f^	8.470^a–f^
ACC44	102.01^a–h^	72.99^e–h^	29.07^bcd^	19.36^cd^	5.518^b–f^	10.470^abc^
ADI	83.01^a–h^	80.99^b–h^	26.74^bcd^	18.36^cd^	5.118^cdef^	11.137^ab^
Baha Necho	119.35^ab^	87.19^a–h^	28.40^bcd^	25.76^bcd^	5.918^a–f^	9.804^abcd^
Baha Zeyit	103.35^a–g^	85.99^a–h^	21.74^bcd^	26.16^bcd^	6.318^a–f^	9.137^a–f^
Borkena	110.68^a–e^	76.79^c–h^	33.07^abc^	27.16^bcd^	8.118^a–f^	10.137^abcd^
Bounji	91.01^a–h^	87.39^a–h^	28.07^bcd^	27.76^bcd^	8.718^a–f^	10.137^abcd^
Gondar 1	104.01^a–f^	76.79^c–h^	14.74^cd^	38.16^ab^	7.718^a–f^	7.470^a–f^
Gumero	110.68^a–e^	71.39^fgh^	27.07^bcd^	44.76^a^	9.518^a–e^	9.470^a–f^
Hirhir	114.01^abcd^	63.99^h^	22.07^bcd^	16.56^cd^	4.518^def^	10.470^abc^
Humera 1	120.68^a^	83.39^a–h^	23.07^bcd^	28.66^bcd^	6.918^a–f^	10.137^abcd^
Setit 1	83.01^a–h^	65.99^fgh^	16.07^cd^	12.56^d^	4.318^df^	10.137^abcd^
Setit 2	74.01^c–h^	64.59^gh^	20.74^bcd^	15.36^cd^	5.518^b–f^	11.470^a^
Zeri Tesfay	116.35^abc^	87.79^a–h^	26.40^bcd^	27.36^bcd^	7.918^a–f^	10.804^abc^
CV	15.3		26		25.1	

PH = plant height, GDFB = ground distance to the first branch, IL = internode length. Means followed by a different letter indicate significant differences at *p* ≤ 0.01.

**Table 5:** Effect of NaN_3_ on different qualitative traits of M2 sesame lines.

MSC		Aberghele (%)	ACC44 (%)	ADI (%)	Baha Necho (%)	Baha Zeyit (%)	Borkena (%)	Bounji (%)	Gondar 1 (%)	Gumero (%)	Hirhir (%)	Humera 1 (%)	Setit 1 (%)	Setit 2 (%)	Zeri Tesfay (%)
Green	Control	37.5	37.5	37.5	37.5	37.5	37.5	37.5	37.5	37.5	37.5	37.5	37.5	37.5	37.5
	Treated	62.5	62.5	62.5	62.5	62.5	62.5	62.5	62.5	62.5	62.5	62.5	62.5	62.5	62.5

*SH*															
Glabrous	Control	37.5						37.5		37.5	100.0	100.0			37.5
	Treated	62.5	100.0	100.0			100.0	62.5	100.0	62.5			100.0		62.5

Weak/sparse	Control		100.0	100.0	100.0	100.0	100.0		100.0				100.0	37.5	
	Treated										100.0	100.0		62.5	

Medium	Control				100.0	100.0									
	Treated	37.5								37.5	100.0	100.0			

*SB*															
Opposite	Control	37.5	37.5	37.5	37.5	37.5	37.5	37.5	37.5	37.5	37.5	37.5	37.5	37.5	37.5
	Treated	62.5	62.5	62.5	62.5	62.5	62.5	62.5	62.5	62.5	62.5	62.5	62.5	62.5	62.5

MSC = main steam color, SH = stem hairiness, SB = stem branch.

**Table 6:** Mean of quantitative traits for each cluster of sesame genotypes in the control.

Cluster	Mean of quantitative traits		
	PH	GDFB	IL
I	80.33	20.44	10.78
II	117.33	23.11	10.33
III	107.07	28.40	9.87
IV	103.22	17.89	8.22

PH = plant height, GDFB = ground distance to the first branch, IL = internode length.

**Table 7:** Mahalanobis distance between clusters in the control.

Clusters	Clusters			
	1	2	3	4
1	—			
2	27.47	—		
3	28.55^*∗*^	26.53	—	
4	33.55^*∗∗*^	43.85^*∗∗*^	12.03	—

N.B. ^*∗*^Significant at 5%, ^*∗∗*^significant at 1%.

**Table 8:** Mean of quantitative traits for each cluster of sesame genotypes treated with NaN_3_.

Cluster	Mean of quantitative traits		
	PH	GDFB	IL
I	64.67	15.27	4.87
II	86.16	27.58	7.24
III	76.80	19.30	5.40
IV	73.10	35.60	8.7

PH = plant height, GDFB = ground distance to the first branch, IL = internode length.

**Table 9:** Mahalanobis distance between clusters of sesame genotypes treated with NaN_3_.

Clusters	Clusters			
	1	2	3	4
1	—			
2	76.91^*∗∗*^	—		
3	19.14	20.58	—	
4	40.07^*∗∗*^	18.09	20.30	—

N.B. ^*∗*^Significant at 5%, ^*∗∗*^significant at 1%.

**Table 10:** Mean of quantitative traits for each cluster of sesame genotypes in combination with their mutants and their control lines.

Cluster	Mean of quantitative traits		
	PH	GDFB	IL
1	102.71	23.31	9.810
2	69.52	16.88	5.080
3	80.36	31.14	7.889

PH = plant height, GDFB = ground distance to the first branch, IL = internode length.

**Table 11:** Mahalanobis distance between clusters of sesame genotypes treated with NaN_3_.

Clusters	Clusters		
	1	2	3
Cluster 1	—		
Cluster 2	48.58^*∗∗*^	—	
Cluster 3	24.36^*∗∗*^	11.71^*∗∗*^	—

N.B. ^*∗*^Significant at 5%, ^*∗∗*^significant at 1%.

## Data Availability

Qualitative and quantitative data of this manuscript are available with the first author.
